# *PON1*, *APOE* and *SDF-1* Gene Polymorphisms and Risk of Retinal Vein Occlusion: A Case-Control Study

**DOI:** 10.3390/genes15060712

**Published:** 2024-05-30

**Authors:** Antonios Ragkousis, Dimitrios Kazantzis, Ilias Georgalas, Panagiotis Theodossiadis, Christos Kroupis, Irini Chatziralli

**Affiliations:** 1 2nd Department of Ophthalmology, Attikon University Hospital, National and Kapodistrian University of Athens, 12462 Athens, Greece; dkaza91@yahoo.gr (D.K.); patheo@med.uoa.gr (P.T.); eirchat@yahoo.gr (I.C.); 2 1st Department of Ophthalmology, “G. Gennimatas” General Hospital, National and Kapodistrian University of Athens, 11527 Athens, Greece; igeorgalas@yahoo.com; 3 Department of Clinical Biochemistry, Attikon University Hospital, National and Kapodistrian University of Athens, 12462 Athens, Greece; ckroupis@med.uoa.gr

**Keywords:** PON1, APOE, SDF-1, retinal vein occlusion, genetic polymorphisms, oxidative stress, inflammation

## Abstract

Numerous studies have tried to evaluate the potential role of thrombophilia-related genes in retinal vein occlusion (RVO); however, there is limited research on genes related to different pathophysiological mechanisms involved in RVO. In view of the strong contribution of oxidative stress and inflammation to the pathogenesis of RVO, the purpose of the present study was to investigate the association of inflammation- and oxidative-stress-related polymorphisms from three different genes [*apolipoprotein E (APOE), paraoxonase 1 (PON1) and stromal cell-derived factor 1 (SDF-1)*] and the risk of RVO in a Greek population. Participants in this case-control study were 50 RVO patients (RVO group) and 50 healthy volunteers (control group). Blood samples were collected on EDTA tubes and genomic DNA was extracted. Genotyping of rs854560 (L55M) and rs662 (Q192R) for the *PON1* gene, rs429358 and rs7412 for the *APOE* gene and rs1801157 [*SDF1*-3′G(801)A] for *SDF-1* gene was performed using the polymerase chain reaction–restriction fragment length polymorphism (PCR-RFLP) method. Multiple genetic models (codominant, dominant, recessive, overdominant and log-additive) and haplotype analyses were performed using the SNPStats web tool to assess the correlation between the genetic polymorphisms and the risk of RVO. Binary logistic regression analysis was used for the association analysis between *APOE* gene variants and RVO. Given the multifactorial nature of the disease, our statistical analysis was adjusted for the most important systemic risk factors (age, hypertension and diabetes mellitus). The dominant genetic model for the *PON1* Q192R single nucleotide polymorphism (SNP) of the association analysis revealed that there was a statistically significant difference between the RVO group and the control group. Specifically, after adjusting for age and hypertension, the *PON1* 192 R allele (QR + RR) was found to be associated with a statistically significantly higher risk of RVO compared to the QQ genotype (OR = 2.51; 95% CI = 1.02–6.14, *p* = 0.04). The statistically significant results were maintained after including diabetes in the multivariate model in addition to age and hypertension (OR = 2.83; 95% CI = 1.01–7.97, *p* = 0.042). No statistically significant association was revealed between the other studied polymorphisms and the risk of RVO. Haplotype analysis for *PON1* SNPs, L55M and Q192R, revealed no statistically significant correlation. In conclusion, *PON1* 192 R allele carriers (QR + RR) were associated with a statistically significantly increased risk of RVO compared to the QQ homozygotes. These findings suggest that the R allele of the *PON1* Q192R is likely to play a role as a risk factor for retinal vein occlusion.

## 1. Introduction

Retinal vein occlusion (RVO), either central RVO (CRVO) or branch RVO (BRVO), is the second most common retinal vascular disorder after diabetic retinopathy, affecting more than 28 million people worldwide with a comparable prevalence between females and males. It is estimated that by 2050, the number of affected individuals in Europe will rise by more than 20% [1,2]. Age is a major risk factor as the incidence increases significantly in people over 50 years old; however, RVO can also occur in younger patients [3]. Depending on the anatomical location of the occlusion and the involvement or not of the macula, the presenting clinical symptoms vary from asymptomatic to unilateral painless visual loss. It is worth noting that BRVO causes less visual impairment and has a better prognosis compared to CRVO, although it is five-fold more prevalent than CRVO [4]. RVO can be further classified as ischemic and nonischemic depending on the extent of capillary nonperfusion areas [5].

The aetiopathogenesis of RVO is multifactorial; however, the exact pathogenetic mechanism remains elusive. It has been suggested that compression and mechanical stenosis of the retinal vein caused by an atherosclerotic artery and local inflammation induced by venous stasis are the predominant pathogenetic events in most cases [6,7]. Systemic hypertension, diabetes mellitus and open-angle glaucoma have been found to be strong independent risk factors associated with all types of RVO. Other systemic risk factors, including cardiovascular disease, hyperlipidemia, an increased body mass index and higher serum levels of the inflammation-related α2 globulin were also implicated in BRVO. In addition, hematological disorders that lead to hyperviscosity may predispose a person to RVO [3,5,6,8].

Both CRVO and BRVO can cause significant visual impairment secondary to macular edema or retinal ischemia, while ocular neovascularisation can be a devastating complication, especially in the case of anterior segment involvement (neovascular glaucoma). There are two main pathophysiological pathways in RVO: (1) inner blood–retinal barrier damage promoted by the release of inflammatory cytokines and the oxidative stress-generated reactive oxygen species; and (2) tissue hypoxia due to vessel closure, resulting in increased expression of vascular endothelial growth factor (VEGF) [6,7,9,10].

So far, genetic research on RVO has been focused on thrombophilia-related genes, which encode proteins that are involved in the coagulation pathway, with the results being controversial [11]. Based on the lack of clear association, current clinical practice guidelines do not include thrombophilia screening in the routine diagnostic workup of patients presenting with vein occlusion. The identification of novel genetic susceptibility loci for RVO may contribute to a deeper understanding of the disease and provide new diagnostic strategies and therapeutic targets.

In light of the above-described pathophysiology of RVO and the crucial role of oxidative stress and inflammation, the purpose of this pilot study was to investigate the potential association of five genetic polymorphisms from three distinct genes relevant to oxidative stress and inflammation [*apolipoprotein E (APOE), paraoxonase 1 (PON1) and stromal cell-derived factor 1 (SDF-1)*] with RVO.

## 2. Materials and Methods

### 2.1. Subjects

The population of our case-control study consisted of 50 consecutive patients diagnosed with RVO and 50 healthy controls recruited at the 2nd Department of Ophthalmology of the National and Kapodistrian University of Athens, at Attikon University Hospital, in Athens. All participants were native Greek and unrelated (no consanguinity). The study protocol was in adherence to the ethical principles of the Declaration of Helsinki and approved by the Institutional Review Board of Attikon University Hospital (Reference number 290/2020). Clinical diagnosis of RVO was based on retinal examination findings of flame-shaped and dot-blot hemorrhages, retinal vein dilatation and tortuosity, with or without cotton wool spots and optic disc hyperemia. Spectral-domain optical coherence tomography (SD-OCT) as well as Fundus fluorescein angiography (FFA) were used for confirmation of the diagnosis.

A comprehensive eye examination, which included best-corrected visual acuity measurement, Goldmann applanation tonometry, slit lamp biomicroscopy, gonioscopy, fundoscopy following dilation of the pupils and SD-OCT of the macula, was conducted for all the study subjects.

Exclusion criteria were uncontrolled glaucoma with intraocular pressure greater than 30 mmHg, ocular inflammation, ocular trauma, prior intraocular surgical operation performed within the previous six months, corneal disease, inadequate fundal view due to media opacities and any other ocular disease affecting the retina or the optic nerve.

Demographic data (age, sex) and the medical history of the study participants were recorded. The diagnosis of systemic arterial hypertension included a history of antihypertensive medication or repeated, on 2 or more occasions, resting blood pressure measurement readings ≥ 140/90 mmHg. Diabetes mellitus was considered present in individuals who were under treatment with antidiabetic agents.

### 2.2. Genotyping

Five single nucleotide polymorphisms (SNPs) of three different genes, namely *APOE* rs429358 and rs7412, *PON1* rs854560 and rs662 and *SDF-1* rs1801157, were selected to be included in the study after a meticulous literature review on RVO genetics because of their relationship with inflammation and oxidative stress.

Blood specimens, obtained from all the 100 study participants by peripheral venous puncture, were collected into ethylenediaminetetraacetic acid (EDTA) tubes. Genomic DNA was isolated from whole blood using a Macherey-Nagel NucleoSpin blood mini kit following the manufacturer’s instructions and stored at −20 °C. The genotyping for all the selected SNPs was determined using the polymerase chain reaction—restriction fragment length polymorphism (PCR—RFLP) method with sequence-specific primers. The Eppendorf 5331 Gradient MasterCycler Thermal Cycler (Eppendorf, Hamburg, Germany) was the laboratory instrument used for the PCR. A 2X Promega GoTaq G2 Hot Start Green Master Mix (Promega, Madison, WI, USA), which contained a thermostable hot-start polymerase, deoxyribonucleotide triphosphates (dNTPs), MgCl_2_ and reaction buffers, was used for the PCR for the *PON1* and *SDF-1* polymorphisms in a total volume of 25 μL. A 2X QIAGEN HotStarTaq Plus Master Mix Kit (QIAGEN, Hilden, Germany), which contained DNA polymerase, QIAGEN PCR Buffer, MgCl_2_ and dNTPs, was used for *APOE* in a total volume of 17.5 μL After amplification, digestion of the PCR products by restriction enzymes and agarose gel electrophoresis 4%, the DNA fragments were stained with ethidium bromide to be visualized by ultraviolet fluorescence. Incubation of the restriction digestion reactions in a water bath at 37 °C for 4 h for *APOE* polymorphisms and for 3 h for *PON1* and *SDF-1* polymorphisms was performed to provide optimal conditions for enzyme activity; 10X CutSmart Buffer was used for *PON1* polymorphisms and M Buffer was used for *APOE* and *SDF-1* polymorphisms.

The genotyping analysis was performed at the laboratory of the Department of Clinical Biochemistry and Molecular Diagnostics of the National and Kapodistrian University of Athens, at Attikon University Hospital, in Athens.

The two *APOE* SNPs, rs429358 and rs7412, derived from two amino acid substitutions at positions 112 (cysteine to arginine) and 158 (arginine to cysteine) in exon 4, respectively. The combination of these two *APOE* SNPs gives rise to three *APOE* haplotypes, which are widely referred to as alleles, namely E2, E3 and E4. The primers used for *APOE* genotyping were upstream primer F: 5′-ACAGAATTCGCCCCGGCCTGGTACAC-3′ and downstream primer R: 5′-TAAGCTTGGCACGGCTGTCCAAGGA-3′. PCR thermal cycling conditions were as follows: initial denaturation at 95 °C for 5 min, followed by 35 cycles including denaturation at 95 °C for 1 min, primers annealing at 60 °C for 1 min and subsequent extension at 72 °C for 1 min; a final extension of 5 min duration at 72 °C was also performed. After amplification, the 244 base pair (bp) PCR product was cleaved by the HhaI restriction enzyme (New England BioLabs, Ipswich, MA, USA), resulting in unique combinations of HhaI fragment sizes for each one of the *APOE* E2, E3 and E4 alleles. Specifically, E2 was characterized by the presence of 91 bp and 83 bp fragments, E3 by 91 bp, 48 bp and 35 bp fragments and E4 by 72 bp, 48 bp and 35 bp fragments [12].

The *SDF-1* rs1801157 polymorphism contains a G to A transition at position 801 in the 3′-untranslated region (3′-UTR) of exon 10 and, for this reason, it is also written as *SDF1*-3′G(801)A. The sequences of the primers were the following: 5′-GAGTCAACCTGGGCAAAGCC-3′ (upstream primer) and 5′-AGCTTTGGTCCTGAGAGTCC-3′ (downstream primer). The PCR cycling conditions were the following: initial denaturation at 94 °C for 4 min, followed by 34 cycles including denaturation at 94 °C for 1 min, primers annealing at 50 °C for 1 min and subsequent extension at 72 °C for 1 min; a final extension at 72 °C for 5 min was also performed. The MspI restriction enzyme (New England BioLabs, Ipswich, MA, USA) was used to digest the amplified 302 bp PCR products. The presence of *SDF1*-3′(801)G allele results in 202 bp and 100 bp bands, whereas the *SDF1*-3′(801)A allele gives one uncut 302 bp band as there is no restriction site after the G to A substitution. The heterozygous *SDF1*-3′(801)GA genotype was revealed by the presence of all three 100, 202 and 302 bp bands [13].

For the PON1 gene, the rs854560 SNP was a leucine → methionine substitution at position 55 of exon 3 (abbreviated as L55M) and the rs662 SNP was a glutamine → arginine substitution at position 192 of exon 6 (abbreviated as Q192R).

**Figure 1:**
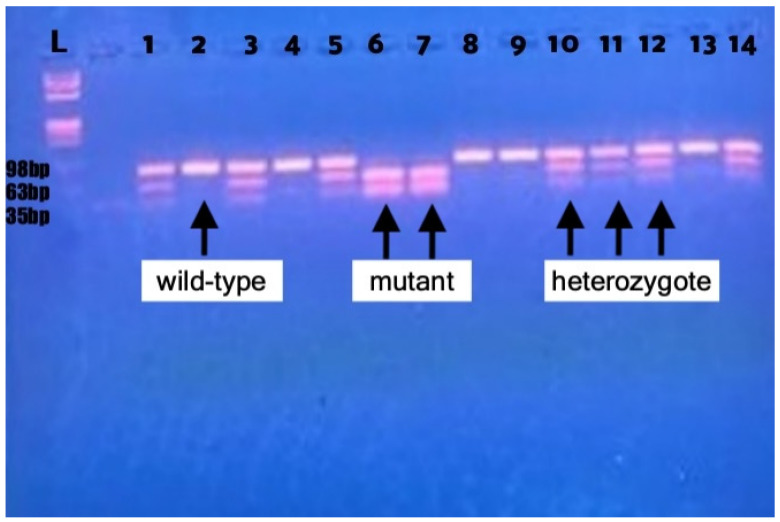
PCR-RFLP assays of *paraoxonase 1 (PON1)* Q192R after digestion of the amplified DNA by the AlwI restriction enzyme. Lanes 2, 4, 8, 9, 13—wild-type QQ genotype; Lanes 6, 7—mutant RR genotype; Lanes 1, 3, 5, 10, 11, 12, 14—heterozygous QR genotype. Lane L represents the φX174 (phiX174) Hae III digest DNA molecular weight marker ladder. The size of the restriction fragments is written with black letters on the left side of the figure.

The primers used for L55M were the following: 5′-GCTCTAGTCCATCAATTTAAAACAAA-3′ (forward) and 5′-TGGGTATACAGAAAGCCTAAGTGA-3′(reverse). For Q192R, the forward primer was 5′-TATTGTTGCTGTGGGACCTGAG-3′ and the reverse was 5′-CACGCTAAACCCAAATACATCTC-3′. The PCR thermocycling steps for *PON1* polymorphisms were as follows: initial denaturation at 94 °C for 4 min, followed by 40 cycles including denaturation at 94 °C for 1 min, primers annealing at 56 °C for 1 min and subsequent extension at 72 °C for 1 min; a final extension stage of 5 min duration at 72 °C was also performed. The NlaIII and AlwI restriction enzymes (New England BioLabs, Ipswich, MA, USA) were used to cleave the PCR products for the L55M and Q192R polymorphisms, respectively. Regarding the L55M SNP, PCR-RFLP electrophoresis results displaying an uncleaved 394 bp band were classified as homozygous LL genotype (wild-type), results illustrating 124 bp and 245 bp fragments were classified as homozygous MM genotype and results displaying all three 124 bp, 245 bp and 394 fragments were classified as heterozygous LM genotype [14]. For the Q192R genotyping, the homozygous QQ genotype (wild-type) was identified by the presence of a single uncut 98 bp band and the homozygous RR genotype corresponded to a combination of 35 bp and 63 bp fragments; the display of all three 35 bp, 63 bp and 98 bp fragments indicated the heterozygous QR genotype [15]. Figure 1 shows the PCR-RFLP assay (4% agarose gel electrophoresis) of the *PON1* Q192R polymorphism.

### 2.3. Statistical Analysis

The statistical analysis of the data was conducted using the Statistical Package for Social Sciences (SPSS) software version 29.0.2.0 (IBM Corp., Armonk, NY, USA) and the SNPStats web tool (https://www.snpstats.net/start.htm, accessed on 25 March 2024) [16].

The Shapiro–Wilk and Kolmogorov–Smirnov normality tests were used to determine whether age, the only quantitative parameter, was distributed normally. Age was expressed as mean ± standard deviation. Categorical variables were presented as frequency and percentage. An independent samples *t*-test was used for age comparison between the control and RVO groups. A Chi-square (χ^2^) test was used to compare categorical variables between the two groups.

The Hardy–Weinberg equilibrium (HWE) and the allele and genotype frequencies were obtained using SNPStats. An odds ratio (OR) with a 95% confidence interval (CI) was calculated for the codominant, dominant, recessive, overdominant and log-additive genetic models to assess the contribution of the selected polymorphisms to the risk of RVO; both crude and multivariate analyses, adjusting for age, hypertension and diabetes mellitus, were performed. Haplotype analysis for the two *PON1* gene SNPs, Q192R and L55M, was also performed using SNPStats.

For the *APOE* gene, because of its unique characteristic of having 3 alleles-haplotypes, binary logistic regression analysis of SPSS, covarying for age, hypertension and diabetes, was used to estimate odds ratio and confidence interval for both E2 and E4 allele carriers compared to E3E3 homozygosity (E3E3 was set as referent group); the E2E4 genotype was included in the E2 carriers’ group. Binary logistic regression association analysis for each separate *APOE* genotype was also performed.

## 3. Results

The demographic and clinical characteristics of the study subjects are summarised in Table 1. A total of 100 subjects were included in our case-control study: 50 RVO patients (54% males, 46% females) with a mean age of 67.26 ± 9.45 years and 50 controls (56% males, 44% females) with a mean age of 69.84 ± 13.18 years. The two groups were age- and sex-matched. Among the RVO patients, 35 of them (70%) were diagnosed with CRVO and 15 (30%) had BRVO. The frequencies of hypertension and diabetes were statistically significantly higher in the RVO group compared to the control group (*p* = 0.003 and *p* < 0.001, respectively).

The allele and genotype frequencies in the RVO patients and the control group for all the studied polymorphisms are shown in Table 2. All the selected SNPs conformed to the HWE expectations (*p* > 0.05).

The association analysis between the selected polymorphisms and the RVO risk revealed a statistically significant difference between the RVO group and the control group in the dominant genetic model for *PON1* Q192R rs662 polymorphism. Specifically, after adjusting for age and hypertension, the dominant model showed that the R allele (QR and RR genotypes) was statistically significantly associated with increased risk of RVO development compared to the QQ genotype (OR = 2.51; 95% CI = 1.02–6.14, *p* = 0.04). The statistical significance of the result for the R allele of *PON1* Q192R was maintained after including diabetes mellitus in the multivariate analysis in addition to age and hypertension (OR = 2.83; 95% CI = 1.01–7.97, *p* = 0.042). The result of the univariate (crude) association analysis for *PON1* Q192R was not statistically significant.

The other studied SNPs did not appear to be linked with susceptibility to RVO as none of the genetic models of the association analysis demonstrated any statistically significant difference between the RVO patients and the control group.

The crude and multivariate analyses results, including ORs, 95% CIs and *p*-values, of all the genetic models (codominant, dominant, recessive, overdominant and log-additive) for *PON1* L55M, *PON1* Q192R and *SDF1*-3′G(801)A are presented in Table 3, Table 4 and Table 5, respectively.

Additionally, haplotype analysis for the two SNPs of the *PON1* gene, L55M and Q192R, did not detect any association of any haplotype with the RVO risk (Table 6).

With reference to the *APOE* gene, to investigate its potential effect on RVO, we divided the genotypes into the following genotype subgroups: homozygous E3E3, E2 carriers group (E2E2, E2E3, E2E4) and E4 carriers group (E4E4, E3E4). No correlations between these genotype subgroups and RVO were observed in the binary logistic regression analysis after adjusting for age, hypertension and diabetes (Table 7). Also, no association with RVO was found when each APOE genotype was examined separately using binary logistic regression analysis.

## 4. Discussion

It is well established that age, hypertension, diabetes mellitus and open-angle glaucoma are independent risk factors for the development of RVO, either CRVO or BRVO [8]. BRVO has also been linked to several systemic risk factors, including hyperlipidemia, cardiovascular disease, elevated body mass index, elevated serum levels of the inflammation-related protein α2 globulin and hypercoagulable state. Apart from the body mass index, which is closely related to lack of physical activity, other lifestyle factors such as smoking and diet seem to play a role in RVO. Mediterranean and plant-based diets have been shown to reduce the risk for RVO due to their proven anti-inflammatory and anticoagulant effects [17]. Hypertension is considered as the strongest risk factor because it accelerates arterial stiffness (atherosclerosis), leading to mechanical compression of the adjacent vein. This cascade of events causes mechanical lumen narrowing of the thin-walled veins, abnormal blood flow and endothelial damage, resulting in the formation of an endoluminal thrombus and occlusion [18,19]. However, the exact mechanisms involved in the pathogenesis of RVO remain unclear. The three factors of Virchow’s triad, consisting of alterations in blood flow (venous stasis), vascular endothelial injury and hypercoagulable state, are implicated in thrombogenesis, leading to the RVO [3].

Numerous studies have tried to investigate the potential role of genetic polymorphisms associated with thromboembolic risk in RVO. Several polymorphisms of genes that encode proteins of the coagulation pathway have been studied, including *5,1-methylenetetrahydrofolate reductase (MTHFR)* C677T and A1298C, *factor V Leiden* G1691A, *plasminogen activator inhibitor 1 (PAI-1)* 4G/5G, *factor II* (also known as *prothrombin*) G20210A and *vitamin K epoxide reductase complex subunit 1 (VKORC1)* G1639A, showing conflicting results about the association between these thrombophilic mutations and RVO [20,21,22,23,24]. In a recent meta-analysis, Romiti et al. [24] did not observe different prevalences of *factor V Leiden* G1691A, *prothrombin* G20210A, *MTHFR* C677T and *PAI-1* 4G/5G thrombophilic genetic variants between healthy subjects and patients with retinal vascular occlusion, suggesting that screening for these genetic polymorphisms in patients with retinal vein occlusion or retinal artery occlusion is not clinically useful.

*MTHFR* C677T is the most studied genetic polymorphism regarding its potential role as a risk factor for RVO. The *MTHFR* gene is responsible for the production of the key regulatory enzyme MTHFR, whose activity determines the plasma homocysteine levels. Several case-control studies reported an association between the *MTHFR* 677TT genotype and RVO [25,26,27,28,29], while some other studies and meta-analyses did not reveal any association [24,30,31,32,33,34,35].

Some other researchers aimed to evaluate the association of atherosclerosis-related genetic polymorphisms including *angiotensin II type 1 receptor (AGTR1)* A1166C, *angiotensin I–converting enzyme (ACE)* insertion/deletion, *platelet glycoprotein Ia/IIa (GpIa/IIa)* C807T/G873A, *matrix metalloproteinase 2 (MMP2)* 1306C/T, *tissue inhibitors of matrix metalloproteinase 2 (TIMP2)* G418C and *adiponectin* +276 G/T with RVO [36,37,38,39,40,41]. Both Demir et al. [41] and Christodoulou et al. [36] suggested that *adiponectin* +276 T allele carriers and *AGTR1* C allele carriers are likely to be at increased risk for RVO. *MMP2* 1306C/T was found to predispose to ischemia among patients with RVO [42]. The studies investigating the rest of the abovementioned atherosclerosis-related SNPs demonstrated contradictory results.

There are also studies that selected SNPs from genes relevant to inflammation and oxidative stress, similarly to our gene selection approach and assessed their possible effect on the development of RVO. Heme oxygenase-1 is an important antioxidant enzyme that inhibits proinflammatory responses; no association was found between *heme oxygenase-1* −413A>T and RVO [43]. Maier et al. studied multiple SNPs from different cytokines, including *interleukin (IL)1β* −511C>T, *IL1 receptor antagonist (IL1RN)* 1018T>C, *IL4* −584C>T, *IL6* −174G>C, *IL8* −251A>T, *IL10* −592C>A, *IL18* 183A>G, *C-C motif chemokine ligand 5 (CCL5)* −403G>A, *monocyte chemoattractant protein (MCP)-1/CCL2* −2518A>G and *tumor necrosis factor (TNF)-α* −308G>A; however, none of them were implicated in the development of RVO [44].

Apolipoprotein E (APOE) has an antiatherogenic function by serving as a ligand for the low-density lipoprotein (LDL) receptor, thus facilitating the clearance of triglyceride-rich lipoproteins from circulation and the reduction of plasma cholesterol levels [45]. APOE also has known anti-inflammatory effects by suppressing the activation of the classical complement cascade and the release of proinflammatory cytokines from foam cells [46].

It is known that the *APOE* E4 allele is associated with lower plasma APOE levels and with an increased risk for cardiovascular disease. Apart from its atherogenic nature, the E4 allele is also a known strong genetic risk factor for Alzheimer’s disease as the low plasma APOE levels result in decreased brain b-amyloid clearance [47]. Additionally, the E4 allele has been implicated in RVO and nondiabetic retinopathy [48,49].

On the contrary, it is interesting that the E4 allele is linked with a protective role in each of the age-related macular degeneration (AMD) subtypes, which can be explained by the fact that APOE is an abundant constituent of retinal drusen, being expressed locally by Müller cells and retinal pigment epithelium (RPE) [50,51].

In our study, no correlation was found between the E4 allele and the development of RVO, which aligns with the findings of Salomon et al. [25].

Stromal-cell-derived factor 1 (SDF-1) is a homeostatic chemokine, part of the C-X-C motif subfamily (also known as CXCL12). Hypoxia-induced gene expression and upregulation of SDF-1 in endothelial cells lead to inflammation and a subsequent blood–retina barrier breakdown as well as the promotion of angiogenesis. The binding of SDF-1 to its receptor CXCR4 stimulates the release of VEGF, which in turn provokes further expression of SDF-1 in endothelial cells, resulting in a positive feedback loop. The SDF-1/CXCR4 pathway plays the predominant role in the mobilization of hematopoietic stem-cell-derived endothelial progenitor cells in the process of retinal neovascularisation in retinal ischemic diseases [52,53].

Ki-I et al. [54] documented that SDF-1 vitreous levels were statistically significantly higher in patients with RVO complicated with neovascularisation compared to RVO patients without neovascularisation and controls. On the other hand, Zeng et al. [55] found comparable CXCL12 vitreous concentrations between the RVO patients and control subjects. Szigeti et al. reported that the *SDF1*-3′(801)A allele predisposes people to the development of ocular neovascularisation as a complication of RVO [13].

To our knowledge, our study is the first to investigate the potential association between the *SDF1*-3′G(801)A polymorphism and the development of RVO; however, no correlation was found.

Paraoxonase 1 (PON1) is a multifunctional calcium-dependent enzyme, strongly bound to high-density lipoprotein (HDL), which promotes the anti-inflammatory and antioxidant properties of HDL. PON1 is a promiscuous enzyme with different activities as it can hydrolyze various substrates; it has paraoxonase activity, having the ability to hydrolyze paraoxon as well as lactonase, and arylesterase activities that inhibit LDL peroxidation. The antiatherogenic function of PON1 is, therefore, based on its lactonase and arylesterase activities by reducing the proatherogenic oxidized LDL (ox-LDL), as ox-LDL is directly involved in foam cell formation [56,57].

Q192R is the most widely studied PON1 SNP, as it exerts a substrate-dependent effect on the activity. The Q192 allozyme has higher lactonase and arylesterase antiatherosclerotic activities than the 192R isoenzyme, whereas the opposite applies for paraoxonase activity as 192R is more effective in hydrolyzing paraoxon than Q192. On the other hand, L55M is regarded to influence the PON1 concentrations but not the enzymatic activity directly. The L55 allozyme is associated with increased serum PON1 concentrations compared to the 55M due to its greater stability and resistance to proteolysis [58,59,60].

Angayarkanni et al. [61] found that PON1-arylesterase activity was significantly lower in CRVO patients than in the control group, thus implicating oxidative stress and ox-LDL in CRVO. Ortak et al. [62] suggested a protective effect of the LL genotype of L55M polymorphism on the pathogenesis of RVO, while they did not find any effect of Q192R on RVO.

In this study, L55M did not show a correlation with the development of RVO; however, it is noteworthy that in one of our recent studies, we reported that the LL genotype provides better treatment response to intravitreal injections of anti-VEGF drugs [63].

The finding of the present study, that the *PON1* 192 R allele carriers were associated with a statistically significantly increased risk of RVO compared to the QQ homozygotes, is in line with the previous report of Murata et al. [64] that the *PON1* 192R allele was significantly associated with CRVO. Specifically, in our study, the odds ratio for the *PON1* 192 R allele carriers was 2.51 after adjusting for age and hypertension and 2.83 after adjusting for age, hypertension and diabetes mellitus; in other words, the *PON1* 192 R allele carriers were found to have 2.51 times and 2.81 times, respectively, the risk of RVO compared to QQ homozygotes (a 151% and 181% increase in risk, respectively).

The findings of our case-control study and of the relevant case-control studies for the role of the *APOE*, *SDF-1* and *PON1* genes or their encoded proteins in retinal vein occlusion are summarized in Table 8.

The observed discrepancies in the reported results among studies investigating the relationship between RVO and several different genetic polymorphisms could be attributed to the considerable heterogeneity among studies. This heterogeneity is described by ethnic differences and environmental factors, as well as confounding variables, such as age and other clinical risk factors. Therefore, our approach to adjusting our results for clinical characteristics, including age, hypertension and diabetes, as well to excluding people with glaucoma can be considered as a strength of our study. A potential limitation of our study was the relatively small sample size. Additionally, separate analysis of CRVO and BRVO patients was not performed because of the sample size, although the two types of RVO have differences in their pathophysiology. Finally, the findings of our study might not be applicable to other ethnic groups because all the participants in our study were Greek and the prevalence of genetic variants can vary among populations of different ancestries.

## 5. Conclusions

In conclusion, the findings of our pilot case-control study suggest that the R allele of the *PON1* Q192R is likely to be a risk factor for RVO. No evidence of an association between *PON1* L55M, *APOE* and *SDF1*-3′G(801)A was found. Given the multifactorial nature of the disease, future large-scale studies and meta-analyses, which will take into consideration systemic risk factors and variations among ethnic groups, are required to verify the role of *PON1* Q192R SNP in the susceptibility to RVO and explore potential effects of other SNPs on the pathogenesis of RVO. Such studies would not only further elucidate the pathophysiological mechanisms that are implicated in RVO but may also impact clinical practice by determining the suitability of introducing complementary genetic screening as a preventive measure in high-risk patients.

## Figures and Tables

**Table 1:** Demographic and clinical characteristics of the patients with retinal vein occlusion (RVO) versus control volunteers.

	Control Group	RVO Group	*p*
Age (mean ± standard deviation, years)	69.84 ± 13.18	67.26 ± 9.45	0.263 *
Sex, Males/Females (N, %)	28 (56%)/22 (44%)	27 (54%)/23 (46%)	0.841 ^§^
Hypertension (N, %)	15 (30%)	30 (60%)	0.003 ^§^
Diabetes mellitus (N, %)	3 (6%)	20 (40%)	<0.001 ^§^

* *p*-value calculated using independent samples *t*-test; ^§^ *p*-value calculated using the Chi-square (χ^2^) test. The bold font denotes the presence of statistical significance.

**Table 2:** Allelic and genotypic distribution of *PON1* L55M, *PON1* Q192R, *SDF1*-3′G(801)A and *APOE* in RVO patients and control group.

***PON1* L55M**		**Control Subjects, N (%)**	**RVO Patients, N (%)**
Allele	L	62 (62%)	65 (65%)
	M	38 (38%)	35 (35%)
Genotype	LL	21 (42%)	19 (38%)
	LM	20 (40%)	27 (54%)
	MM	9 (18%)	4 (8%)
Allele carriers (either homozygote or heterozygote)	LL + LM	41 (82%)	46 (92%)
	LM + MM	29 (58%)	31 (62%)
***PON1* Q192R**		**Control Subjects**	**RVO Patients**
Allele	Q	79 (79%)	72 (72%)
	R	21 (21%)	28 (28%)
Genotype	QQ	33 (66%)	26 (52%)
	QR	13 (26%)	20 (40%)
	RR	4 (8%)	4 (8%)
Allele carriers (either homozygote or heterozygote)	QQ + QR	46 (92%)	46 (92%)
	QR + RR	17 (34%)	24 (48%)
***SDF1-3′G(801)A***		**Control Subjects**	**RVO Patients**
Allele	G	69 (69%)	73 (73%)
	A	31 (31%)	27 (27%)
Genotype	GG	22 (44%)	25 (50%)
	GA	25 (50%)	23 (46%)
	AA	3 (6%)	2 (4%)
Allele carriers (either homozygote or heterozygote)	GG + GA	47 (94%)	48 (96%)
	GA + AA	28 (56%)	25 (50%)
***APOE***		**Control Subjects**	**RVO Patients**
Allele	E3	84 (84%)	90 (90%)
	E4	10 (10%)	6 (6%)
	E2	6 (6%)	4 (4%)
Genotype	E3E3	36 (72%)	40 (80%)
	E2E3	5 (10%)	4 (8%)
	E3E4	7 (14)	6 (12%)
	E2E4	1 (2%)	0 (0%)
	E4E4	1 (2%)	0 (0%)
	E2E2	0 (0%)	0 (0%)
Allele carriers (either homozygote or heterozygote)	E2 carriers (E2E2, E2E3, E2E4)	6 (12%)	4 (8%)
	E4 carriers (E4E4, E3E4)	8 (16%)	6 (12%)

**Table 3:** Crude and multivariate association analysis between *PON1* L55M and RVO risk.

PON1 L55M		Frequencies		Crude Analysis		Adjusted for Age + Hypertension		Adjusted for Age + Hypertension + Diabetes	
Model	Genotype	Control Group	RVO Group	OR (95% CI)	*p*-Value	OR (95% CI)	*p*-Value	OR (95% CI)	*p*-Value
Codominant	LL	21 (42%)	19 (38%)	1.00	0.21	1.00	0.3	1.00	0.24
	LM	20 (40%)	27 (54%)	1.49 (0.64–3.48)		1.31 (0.53–3.23)		0.82 (0.29–2.33)	
	MM	9 (18%)	4 (8%)	0.49 (0.13–1.86)		0.45 (0.11–1.87)		0.25 (0.05–1.38)	
Dominant	LL	21 (42%)	19 (38%)	1.00	0.68	1.00	0.91	1.00	0.4
	LM + MM	29 (58%)	31 (62%)	1.18 (0.53–2.63)		1.05 (0.45–2.47)		0.65 (0.24–1.77)	
Recessive	LL + LM	41 (82%)	46 (92%)	1.00	0.13	1.00	0.15	1.00	0.1
	MM	9 (18%)	4 (8%)	0.40 (0.11–1.38)		0.39 (0.10–1.48)		0.28 (0.06–1.38)	
Overdominant	LL + MM	30 (60%)	23 (46%)	1.00	0.16	1.00	0.29	1.00	0.81
	LM	20 (40%)	27 (54%)	1.76 (0.80–3.89)		1.58 (0.68–3.66)		1.13 (0.43–2.95)	
Log-additive	-	-	-	0.88 (0.49–1.57)	0.66	0.82 (0.44–1.53)	0.54	0.59 (0.28–1.25)	0.16

**Table 4:** Crude and multivariate association analysis between *PON1* Q192R and RVO risk.

*PON1* Q192R		Frequencies		Crude Analysis		Adjusted for Age + Hypertension		Adjusted for Age + Hypertension + Diabetes	
Model	Genotype	Control Group	RVO Group	OR (95% CI)	*p*-Value	OR (95% CI)	*p*-Value	OR (95% CI)	*p*-Value
Codominant	QQ	33 (66%)	26 (52%)	1.00	0.31	1.00	0.12	1.00	0.13
	QR	13 (26%)	20 (40%)	1.95 (0.82–4.65)		2.61 (1.01–6.75)		2.87 (0.96–8.60)	
	RR	4 (8%)	4 (8%)	1.27 (0.29–5.57)		2.11 (0.42–10.60)		2.69 (0.42–17.30)	
Dominant	QQ	33 (66%)	26 (52%)	1.00	0.15	1.00	**0.04**	1.00	**0.042**
	QR + RR	17 (34%)	24 (48%)	1.79 (0.80–4.01)		**2.51 (1.02–6.14)**		**2.83 (1.01–7.97)**	
Recessive	QQ + QR	46 (92%)	46 (92%)	1.00	1	1.00	0.65	1.00	0.53
	RR	4 (8%)	4 (8%)	1.00 (0.24–4.24)		1.43 (0.30–6.67)		1.79 (0.30–10.66)	
Overdominant	QQ + RR	37 (74%)	30 (60%)	1.00	0.14	1.00	0.062	1.00	0.08
	QR	13 (26%)	20 (40%)	1.90 (0.81–4.43)		2.36 (0.94–5.89)		2.52 (0.88–7.23)	
Log-additive	-	-	-	1.41 (0.76–2.63)	0.27	1.84 (0.93–3.67)	0.075	2.05 (0.94–4.48)	0.067

The bold font denotes the presence of statistical significance.

**Table 5:** Crude and multivariate association analysis between *SDF1*-3′G(801)A and RVO risk.

*SDF1*-3′G(801)A		Frequencies		Crude Analysis		Adjusted for Age + Hypertension		Adjusted for Age + Hypertension + Diabetes	
Model	Genotype	Control Group	RVO Group	OR (95% CI)	*p*-Value	OR (95% CI)	*p*-Value	OR (95% CI)	*p*-Value
Codominant	GG	22 (44%)	25 (50%)	1.00	0.79	1.00	0.8	1.00	0.74
	GA	25 (50%)	23 (46%)	0.81 (0.36–1.81)		1.05 (0.44–2.51)		0.96 (0.35–2.63)	
	AA	3 (6%)	2 (4%)	0.59 (0.09–3.84)		0.53 (0.07–3.91)		0.42 (0.04–3.96)	
Dominant	GG	22 (44%)	25 (50%)	1.00	0.55	1.00	0.96	1.00	0.79
	GA + AA	28 (56%)	25 (50%)	0.79 (0.36–1.73)		0.98 (0.42–2.27)		0.88 (0.33–2.31)	
Recessive	GG + GA	47 (94%)	48 (96%)	1.00	0.65	1.00	0.51	1.00	0.44
	AA	3 (6%)	2 (4%)	0.65 (0.10–4.08)		0.52 (0.07–3.68)		0.42 (0.05–3.85)	
Overdominant	GG + AA	25 (50%)	27 (54%)	1.00	0.69	1.00	0.81	1.00	0.93
	GA	25 (50%)	23 (46%)	0.85 (0.39–1.87)		1.11 (0.47–2.61)		1.05 (0.39–2.78)	
Log-additive	-	-	-	0.79 (0.40–1.55)	0.49	0.90 (0.44–1.83)	0.77	0.81 (0.36–1.82)	0.61

**Table 6:** Haplotype analysis of *PON1* L55M and Q192R SNPs for association with RVO.

Haplotype and Frequencies Estimation						Haplotype Association with RVO					
						Crude Analysis		Adjusted for Age + Hypertension		Adjusted for Age + Hypertension + Diabetes	
L55M	Q192R	Total	Control Group	RVO Group	Cumulative Frequency	OR (95% CI)	*p*-Value	OR (95% CI)	*p*-Value	OR (95% CI)	*p*-Value
L	Q	0.40	0.42	0.39	0.40	1.00	-	1.00	-	1.00	-
M	Q	0.35	0.37	0.33	0.76	1.02 (0.51–2.03)	0.96	1.06 (0.50–2.25)	0.89	0.76 (0.32–1.78)	0.53
L	R	0.23	0.20	0.26	0.99	1.42 (0.64–3.14)	0.39	1.82 (0.76–4.38)	0.18	1.88 (0.72–4.90)	0.2
M	R	0.01	0.01	0.02	1	1.53 (0.08–30.50)	0.78	2.96 (0.16–54.11)	0.47	1.02 (0.05–22.96)	0.99

**Table 7:** Binary logistic regression analysis for association between *APOE* genotype groups and RVO.

APOE	Crude Analysis		Adjusted for Age + Hypertension		Adjusted for Age + Hypertension + Diabetes	
Genotype Group	OR (95% CI) ^†^	*p*-Value	OR (95% CI) ^†^	*p*-Value	OR (95% CI) ^†^	*p*-Value
E3E3	Reference	-	Reference	-	Reference	-
E2 carriers	0.60 (0.16–2.30)	0.456	0.45 (0.11–1.90)	0.275	0.49 (0.09–2.80)	0.424
E4 carriers	0.68 (0.21–2.13)	0.503	0.53 (0.15–1.90)	0.323	0.65 (0.17–2.59)	0.545

^†^ Odds ratio estimate from binary logistic regression analysis.

**Table 8:** Summary of the findings of our case-control study and of the relevant case-control studies for the role of *APOE*, *SDF-1* and *PON1* genes or their encoded proteins in retinal vein occlusion.

Study	Country	Gene Polymorphism/Encoded Protein	Design and Purpose of the Study	Sample Size	Results
Mrad et al. [48]	Tunisia	*APOE* E2, E3, E4 (SNPs: rs429358 and rs7412)	Association between *APOE* main allelic types (E2, E3, E4) and RVO	88 RVO patients, 100 control subjects	E4 allele significantly associated with RVO (Odds ratio = 3.8, CI = 2.1–6.8), whereas E3 is protective against the disease (odds ratio = 0.32, CI = 0.19–0.53). No association between E2 allele and RVO
Salomon et al. [25]	Israel	*APOE* E2, E3, E4	Association between *APOE* main allelic types (E2, E3, E4) and RVO	94 RVO patients, 92 control subjects	Homo- or Heterozygosity for E4 allele is not a risk factor for RVO (odds ratio = 1.32, CI = 0.61–2.84)
Szigeti et al. [13]	Hungary	*SDF1*-3′G(801)A polymorphism	Role of *SDF1*-3′G(801)A polymorphism in the development of ocular neovascularisation (either anterior or posterior segment) as complication in RVO	130 RVO patients, 125 control subjects	*SDF1*-3′(801)A allele predisposes to the development of neovascularisation as complication of RVO (Odds ratio = 2.69, CI = 1.47–4.93)
Ki-I et al. [54]	Japan	SDF-1 (stromal cell-derived factor 1 cytokine) levels in vitreous	Relationship between SDF-1 vitreous concentration and iris and/or angle neovascularisation	20 RVO patients, 9 control subjects	SDF-1 vitreous levels were statistically significantly higher in RVO patients complicated with neovascularisation than RVO patients without neovascularisation and controls
Zeng et al. [55]	China	CXCL12 (C-X-C motif chemokine 12, also known as SDF-1) levels in vitreous	Correlation between chemokines vitreous levels and ischemic RVO	25 RVO patients, 20 control subjects	CXCL12 vitreous levels were comparable between the RVO group and control group
Murata et al. [64]	Japan	*PON1* Q192R polymorphism	Role of *PON1* Q192R polymorphism as risk factor for CRVO	42 CRVO patients, 45 control subjects	*PON1* 192R allele significantly associated with CRVO
Ortak et al. [62]	Turkey	*PON1* Q192R and *PON1* L55M polymorphisms	Association of *PON1* Q192R and *PON1* L55M polymorphisms with RVO	120 RVO patients, 84 control subjects	*PON1* 55LL was found statistically significantly lower in RVO patients compared to control subjects; no association was found between *PON1* Q192R and RVO
Angayarkanni et al. [61]	India	Serum PON1	Correlation between PON1 arylesterase (PON1-ARE) activity and RVO	10 CRVO patients, 20 control subjects	PON1-ARE activity was significantly lower in CRVO patients than in the control group. There was a negative correlation between serum PON-ARE and plasma homocysteine levels
Present study	Greece	*APOE* (E2, E3, E4), *SDF1*-3′G(801)A polymorphism, *PON1* Q192R and *PON1* L55M polymorphisms	Association of *PON1, APOE* and *SDF-1* gene polymorphisms with risk of retinal vein occlusion	50 RVO patients and 50 control subjects	*PON1* 192R allele significantly associated with RVO (OR = 2.83, CI = 1.01–7.97) No statistically significant association with RVO was found for *APOE*, *SDF1*-3′G(801)A and *PON1* L55M

## Data Availability

Data will be available upon request.
